# MiR-375 Promotes Redifferentiation of Adult Human β Cells Expanded In Vitro

**DOI:** 10.1371/journal.pone.0122108

**Published:** 2015-04-13

**Authors:** Gili Nathan, Sharon Kredo-Russo, Tamar Geiger, Ayelet Lenz, Haggai Kaspi, Eran Hornstein, Shimon Efrat

**Affiliations:** 1 Department of Human Molecular Genetics and Biochemistry, Sackler School of Medicine, Tel Aviv University, Tel Aviv, Israel; 2 Department of Molecular Genetics, Weizmann Institute of Science, Rehovot, Israel; University of Torino, ITALY

## Abstract

In-vitro expansion of β cells from adult human pancreatic islets could provide abundant cells for cell replacement therapy of diabetes. However, proliferation of β-cell-derived (BCD) cells is associated with dedifferentiation. Here we analyzed changes in microRNAs (miRNAs) during BCD cell dedifferentiation and identified miR-375 as one of the miRNAs greatly downregulated. We hypothesized that restoration of miR-375 expression in expanded BCD cells may contribute to their redifferentiation. Our findings demonstrate that overexpression of miR-375 alone leads to activation of β-cell gene expression, reduced cell proliferation, and a switch from N-cadherin to E-cadherin expression, which characterizes mesenchymal-epithelial transition. These effects, which are reproducible in cells derived from multiple human donors, are likely mediated by repression of PDPK1 transcripts and indirect downregulation of GSK3 activity. These findings support an important role of miR-375 in regulation of human β-cell phenotype, and suggest that miR-375 upregulation may facilitate the generation of functional insulin-producing cells following ex-vivo expansion of human islet cells.

## Introduction

Beta-cell replacement by regeneration or transplantation is considered a promising therapy for diabetes. Transplantation is greatly hindered by shortage of human islet donors. In-vitro expansion of β cells from adult human pancreatic islets could provide abundant insulin-producing cells for transplantation, however induction of islet cell replication in culture leads to loss of β-cell phenotype, in a process resembling epithelial-mesenchymal transition (EMT) [1–3]. Expanded human β-cell-derived (BCD) cells, which constitute ~40% of cells in islet cell cultures [2], maintain open chromatin structure at β-cell genes [4], and can be redifferentiated in response to a combination of soluble factors termed Redifferentiation Cocktail (RC) [5]. These factors include activin A, exendin-4, nicotinamide, and high glucose concentrations, which have been shown to promote β-cell differentiation, in serum-free medium supplemented with B27 and insulin-transferrin-selenium. However, RC treatment leads to redifferentiation of only part of BCD cells. In search for improved redifferentiation approaches, we analyzed changes in microRNAs (miRNAs) during BCD cell dedifferentiation. miRNAs are endogenous short noncoding RNAs which bind to the 3′-untranslated regions of target mRNAs and act as negative regulators of gene expression [6]. miRNAs play important roles in regulation of islet development, β-cell differentiation and function [7,8], and human diabetes [9]. Among the miRNAs highly expressed in islets, miR-375 has been shown to be required for normal mouse glucose homeostasis [10] and zebrafish β-cell development [11], and expressed at high levels during human islet development [12], as well as in mature islets [13,14]. Using miRNA microarray analyses we identified miR-375 as one of the miRNAs greatly downregulated during BCD cell proliferation in vitro. We hypothesized that restoration of miR-375 expression in expanded BCD cells may contribute to their redifferentiation. Our findings demonstrate that overexpression of miR-375 alone activates BCD cell redifferentiation by affecting multiple targets.

## Materials and Methods

### Ethics statement

This study was conducted according to the principles expressed in the Declaration of Helsinki. The Institutional Review Boards of the following medical centers, which provided human islets, each provided approval for the collection of samples and subsequent analysis: University of Geneva School of Medicine; San Raffaele Hospital, Milan; Faculty of Medicine, Lille 2 University; Massachusetts General Hospital; Washington University; University of Pennsylvania; Scharp/Lacy Institute; University of Illinois; University of Wisconsin; University of Miami; Southern California Islet Consortium. All donors provided written informed consent for the collection of all samples and subsequent analysis.

### Cell culture

Human islets were received 2–4 days following isolation from individual donors (Table 1). Islets were dissociated into single cells and cultured in CMRL 1066 medium containing 5.6 mM D-glucose and supplemented with 10% FCS (HyClone), 100 U/ml penicillin, 100 μg/ml streptomycin, 100 μg/ml gentamycin, and 5 μg/ml amphotericine B (Biological Industries) (growth medium) as described [1]. The cultures were refed twice a week and split 1:2 once a week. For redifferentiation, expanded cells in passages 5–7 were trypsinized and seeded in ultra-low attachment plates with Redifferentiation Cocktail (RC) for 4–8 days as previously described [5]. The medium was replaced every two days.

**Table 1:** Islet donors used in the study.

Donor no.	Sex	Age	BMI	Islet purity (%)
1	m	47	33.2	90
2	f	49	27.1	90
3	f	39	21.9	90
4	f	42	32.5	93
5	m	39	27.4	98
6	m	44	24.7	99
7	m	21	33.8	85
8	f	22	30.0	85
9	f	51	39.3	80
10	m	45	31.6	75
11	m	26	24.0	90
12	f	57	29.0	90
13	m	57	34.3	75
14	m	56	33.8	60
15	m	20	19.5	70
16	m	55	30.6	95
17	m	47	36.1	85
18	m	21	32.3	92
19	m	21	37.0	90
20	m	27	20.2	85
21	m	62	31.8	99
22	m	44	24.7	99
23	f	47	20.6	90
24	m	43	34.7	80
25	m	63	24.5	85
26	f	51	21.2	85
27	m	45	26.7	85
28	f	51	29.7	85
29	m	41	21.9	70
30	f	61	25.1	85
31	m	31	23.1	90
32	f	60	28.0	75
33	m	55	22.4	70
34	f	38	24.0	75
35	f	50	21.5	80
36	f	32	26.9	80
37	f	63	23.4	70
38	m	64	24.2	80
39	f	46	33.0	80
40	m	55	27.0	80
41	f	47	21.9	90
42	f	54	28.5	75
43	m	32	25.7	70
44	m	54	23.0	90
45	f	62	25.4	80
46	f	38	20.8	95
47	f	62	27.1	95
48	f	54	29.4	83
Mean±SE		46±7	27±4	84±12

### β-cell labeling and sorting

RIP-Cre/ER and pTrip–loxP-NEO-STOP-loxP-eGFP lentiviruses [3] were used for lineage tracing. Virus production, cell infection, and tamoxifen treatment were previously described [3]. eGFP-labeled cells were sorted using a FACS Aria sorter (BD Biosciences) as described [2].

### Virus production and cell infection

Pre-mmu-miR-375 was subcloned into pBABE-Bleomycin vector and co-transfected into human embryonic kidney 293T cells for virus production with the Ampo-helper packaging plasmid. The medium was replaced 6h post-transfection, and the virus was harvested 24h later and used fresh. 10^6^ human islet cells were plated in 14-cm culture dishes in growth medium for 24h. Cells were infected at MOI of 3:1 in medium containing 8 μg/ml polybrene (Sigma-Aldrich) for 6h. The infection was repeated two more times in the following two days. Selection of bleomycin-resistant cells was initiated 2–3 days later with 4 μg/ml bleomycin for 5 days. Following selection (total of 10 days from the first infection), the cells were harvested for analysis. *PDPK1* shRNA lentivirus vectors (TRCN-1476, TRCN-1478, TRCN-1479, and TRCN-1413) were obtained from Sigma-Aldrich. Virus was produced in 293T cells following co-transfection with the pCMVdR8.91 and pMD2.G packaging plasmids using FUGENE6 (Roche Diagnostics) or TransIT-LT1 (Mirus). The culture medium was harvested 36, 60 and 84h later, filtered through 0.45-μm filter, and stored at -80°C. Cells were infected at MOI 3:1 in growth medium containing 8 μg/ml polybrene overnight. Selection was initiated 48h later with 4 μg/ml puromycin for 3 days. Following selection (a total of 5 days from infection), cells were harvested for analysis.

### MicroRNA arrays

miRNAs were isolated using Ambion mirVana miRNA Isolation Kit (Life Technologies, Carlsbad, CA). RNA was fluorescently-labeled with the mirVana miRNA Labeling Kit (Life Technologies, Carlsbad, CA), and samples were hybridized to an expression array printed in the Whitehead Institute Core Facility, originally described by Baskerville and Bartel [15]. Data was normalized to median hybridization intensity and analyzed using Genepix pro 4000b Axon and JMP statistical software.

### miRNA in-situ hybridization

Islets were fixed overnight with 4% PFA in PBS at 40°C, incubated in 30% sucrose in PBS for 48 hours at 40°C, embedded in OCT, and snap-frozen. Nine-μm sections were cut using a CM3050S cryostat (Leica). Cells were spotted on slides as described above. Slides were hybridized with Dig-labeled Linked Nucleic Acid probes hsa-miR-375 (38181–15 LNA, Exiqon) and scramble-miR (99004–15 LNA) overnight at 57–58°C. Following washes, slides were incubated with amplification reagent (Perkin-Elmer) to visualize peroxidase activity as previously described [16].

### qPCR analysis

Total RNA was extracted using ZR RNA MiniPrep kit (Zymo Research) or TRIzol (Sigma-Aldrich) with DNase I (Thermo Scientific). cDNA was prepared using High Capacity cDNA RT Kit (Applied Biosystems). qPCR was carried out using ABsolute blue qPCR Mix (Thermo Scientific) or FastStart Universal Probe Library Master Mix (Roche) in a 7300 real-time PCR instrument (Applied Biosystems). The results were normalized to transcripts of TATA-box-binding protein (TBP) and human large ribosomal protein (RPLPO). Table 2 lists primer sequences designed for Universal Probe Library (Roche). All reactions were performed with annealing at 60°C for 40 cycles. For undetectable transcripts, the cycle number was set to 40 for comparisons. cDNA for miRNA analyses was prepared and analyzed using Taqman MicroRNA Assay kit (Applied Biosystems) according to the manufacturer, with primers listed in Table 3.

**Table 2:** Primers for qRT-PCR analyses.

Gene	Sense primer	Antisense primer	Ref-seq
*CDKN1A*	CCGAAGTCAGTTCCTTGTGG	CATGGGTTCTGACGGACAT	NM_000389.4
*ECAD*	GCCGAGAGCTACACGTTCA	GACCGGTGCAATCTTCAAA	NM_004360.3
*GCG*	GTACAAGGCAGCTGGCAAC	TGGGAAGCTGAGAATGATCTG	NM_002054.2
*HNF1B*	CACCAACATGTCTTCAAGTAAACAG	TTGTTGCGCACGAAGTAAGT	NM_000458.2
*IAPP*	TTACCAAATTGTAGAGGCTTTCG	CCCTGCCTCTATACACTCACTACC	NM_000415.2
*INS*	AGGCTTCTTCTACACACCCAAG	CACAATGCCACGCTTCTG	NM_000207.2
*MAFA*	AGCGAGAAGTGCCAACTCC	TTGTACAGGTCCCGCTCTTT	NM_201589.2
*MAFB*	AGGGAAGCTGCCAAGCTC	ATTTGACCATAAGACAAGGCTGT	NM_005461.3
*MTPN*	GGAGACTTGGATGAGGTGAAAG	CACCTTCTAGTGTCCGGTTGA	NM_001128619
*NCAD*	CTCCATGTGCCGGATAGC	CGATTTCACCAGAAGCCTCTAC	NM_001792.3
*NEUROD1*	CTGCTCAGGACCTACTAACAACAA	GTCCAGCTTGGAGGACCTT	NM_002500.2
*NKX6*.*1*	CGTTGGGGATGACAGAGAGT	CGAGTCCTGCTTCTTCTTGG	NM_006186.2
*NOTCH2*	GGCAGACTGGTGACTTCACTT	CTCTCACAGGTGCTCCCTTC	NM_024408.2
*PAX4*	CAGGAGGACCAGGGACTACC	GAGCCACTATGGGGAGTGAG	NM_006193.2
*PAX6*	TGCTCCGGCATGAAATATACTA	GTCTCCAAATGTGCAGCAAC	NM_000280.3
*PDX1*	CACATCCCTGCCCTCCTAC	GAAGAGCCGGCTTCTCTAAAC	NM_000209
*PPY*	TCTAGTGCCCATTTACTCTGGAC	GCAGGTGGACAGGAGCAG	NM_002722.3
*RPLPO*	TCTACAACCCTGAAGTGCTTGAT	CAATCTGCAGACAGACACTGG	NM_001002.3
*SST*	ACCCCAGACTCCGTCAGTTT	ACAGCAGCTCTGCCAAGAAG	NM_001048.23
*TBP*	CGGCTGTTTAACTTCGCTTC	CACACGCCAAGAAACAGTGA	NM_003194
*VIM*	GTTTCCCCTAAACCGCTAGG	AGCGAGAGTGGCAGAGGA	NM_003380.3

**Table 3:** Primers for miRNA analyses.

Gene	Sequence	Assay no.
hsa-miR-375	UUUGUUCGUUCGGCUCGCGUGA	000564
hsa-miR-7	UGGAAGACUAGUGAUUUUGUUGU	000268
hsa-miR-30a-5p	UGUAAACAUCCUCGACUGGAAG	000174
hsa-miR-30c	UGUAAACAUCCUACACUCUCAGC	000149
hsa-miR-30d	UGUAAACAUCCCCGACUGGAAG	000420
hsa-miR-200a	UAACACUGUCUGGUAACGAUGU	000502
hsa-miR-200b	UAAUACUGCCUGGUAAUGAUGA	002251
hsa-miR-200c	UAAUACUGCCGGGUAAUGAUGGA	002300
hsa-miR-24	UGGCUCAGUUCAGCAGGAACAG	000402

### Immunoblotting

Total protein was extracted from cells by incubation with a lysis buffer containing 0.5% NP-40 and protease inhibitor cocktail for 10 min. 20–25 μg protein were resolved by SDS-PAGE and electroblotted onto Immobilon-P 0.45-μm membrane (Millipore), followed by incubation overnight at 4°C with primary antibody (Table 4). The bound antibody was visualized with the appropriate horseradish peroxidase-conjugated anti-IgG (Jackson Immunoresearch) and SuperSignal West Pico chemiluminescent substrate (Pierce). Quantification was done using TINA 2.0 software.

**Table 4:** Antibodies used in immunoblotting.

Species	Antigen	Dilution	Manufacturer
Rabbit	AKT	1:1000	Cell Signaling
Rabbit	Phospho-AKT (Thr308)	1:1000	Cell Signaling
Rabbit	β-CATENIN	1:0000	Abcam
Rabbit	GSK-3α	1:200	Santa Cruz
Mouse	Phospho-GSK-3α (Ser21)	1:1000	Cell Signaling
Rabbit	Phospho-GSK-3α/β (Ser21/9)	1:1000	Cell Signaling
Mouse	Phospho-GSK-3α/β (Ser279/216)	1:500	Millipore
Mouse	GSK-3β	1:2500	BD
Rabbit	Phospho-GSK-3β (Ser9)	1:1000	Cell Signaling
Mouse	HSC70	1:1000	Santa Cruz
Rabbit	MAFA	1:1000	Abcam
Rabbit	PDPK1	1:1000	Cell Signaling
Goat	PDX1	1:200	R&D

### Immunofluorescence and cell proliferation analyses

Cells were trypsinyzed, spotted on slides using a Shandon Cytospin4 centrifuge (Thermo Scientific), and fixed for 15 min at RT in 4% paraformaldehyde (PFA). Slides were blocked for 30 min in PBS containing 1% BSA, 5% fetal goat serum and 0.2% saponin (blocking buffer). Slides were incubated overnight at 4°C with primary antibodies diluted in blocking buffer as follows: rat anti-human C-peptide (1:1000; BCBC); mouse anti-human PDX1 (1:500; R&D); rabbit anti-eGFP (1:1000; Life Technologies); mouse anti-β-catenin (1:200; Cell Signaling); and mouse anti-Ki67 (1:200; Zymed). Slides were washed in PBS with 0.1% Tween 20 (Sigma) and incubated with Alexa fluorophore-conjugated secondary antibodies (1:1000; Life Technologies). Nuclei were stained with DAPI (Abcam). Images were taken using a Leica SP5 confocal microscope.

### Cell apoptosis assay

Apoptotic cells were detected by TUNEL staining using the In Situ Cell Death Detection Kit (Roche).

### Proteomics

Cell pellets were solubilized in a buffer containing 6 M urea and 2 M thiourea in 50 mM Tris-HCl pH 7.5. Following protein reduction (1 mM DTT) and alkylation (5 mM iodoacetamide), proteins were digested with trypsin overnight at room temperature. Resulting peptides were purified on C18 tips. Liquid-chromatography mass-spectrometric analysis was performed on the EASY-nLC1000 UHPLC coupled to the Q-Exactive mass spectrometer (Thermo Scientific). Cells from each donor and treatment were analyzed in two technical replicates. MS files were analyzed in MaxQuant with an FDR threshold of 1% on the peptide and protein levels.

### GSK-3 inhibition

Cells were incubated with SB-216763 (Sigma) at 3 or 6 μM for 48h and then harvested for analysis.

### Statistical analyses

Significance of qPCR and immunoblotting data was determined by two-tailed t-test. To approach a normal distribution, logarithmic transformation was preformed. Significance of immunofluorescence cell counts was determined by χ2 test.

## Results

### Changes in miRNA expression during BCD cell expansion in vitro

**Fig 1:**
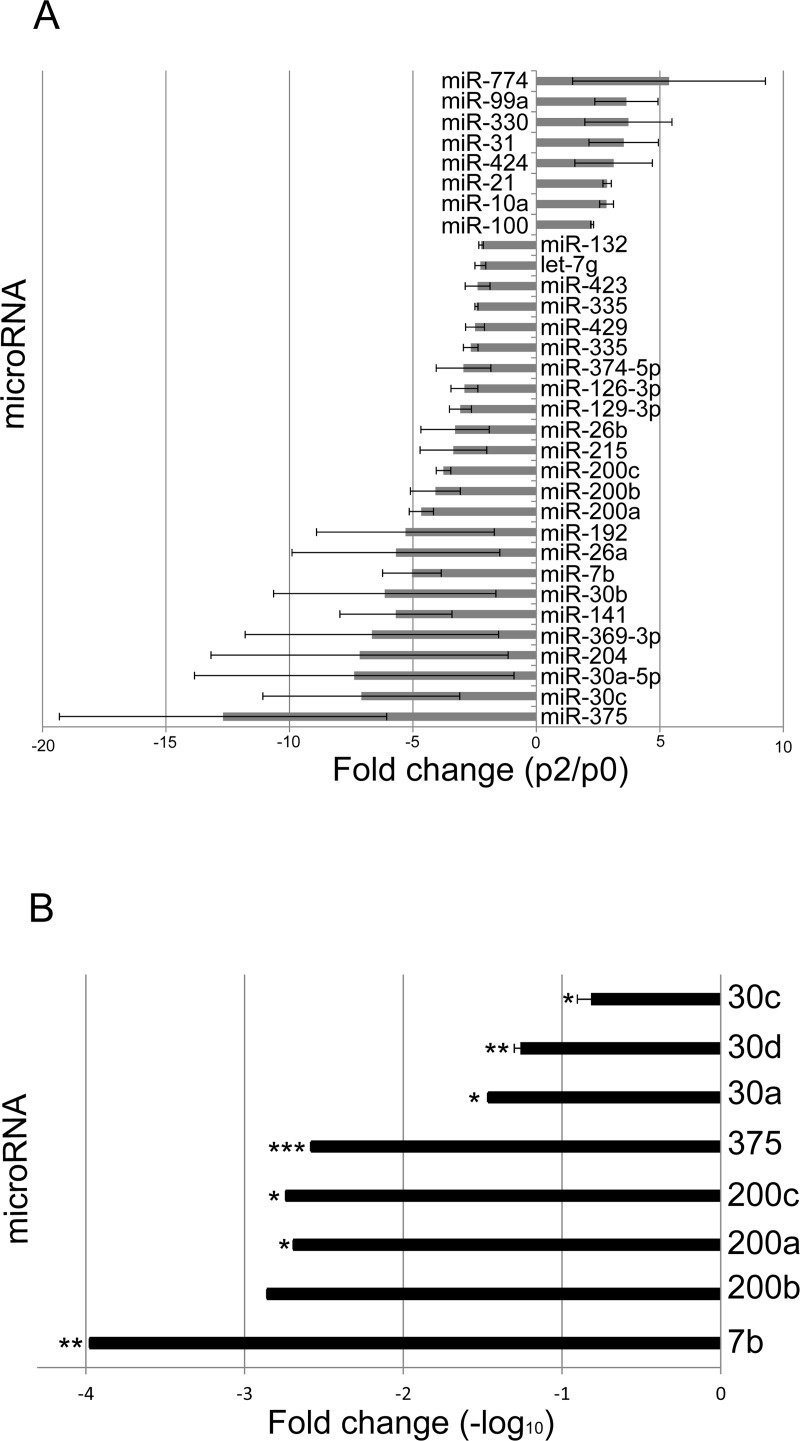
Changes in miRNA expression during BCD cell expansion in vitro. A. miRNA microarray analysis. Values represent the ratio between levels in expanded islet cells at passage 2 and isolated human islets. Data are mean of results from 2 donors. B. qRT-PCR analysis of RNA extracted from sorted GFP^+^ BCD cells at passage 2. Values are mean±SE, relative to islets (n = 3 donors). *p ≤0.05; **p≤0.01; ***p≤0.001.

**Fig 2:**
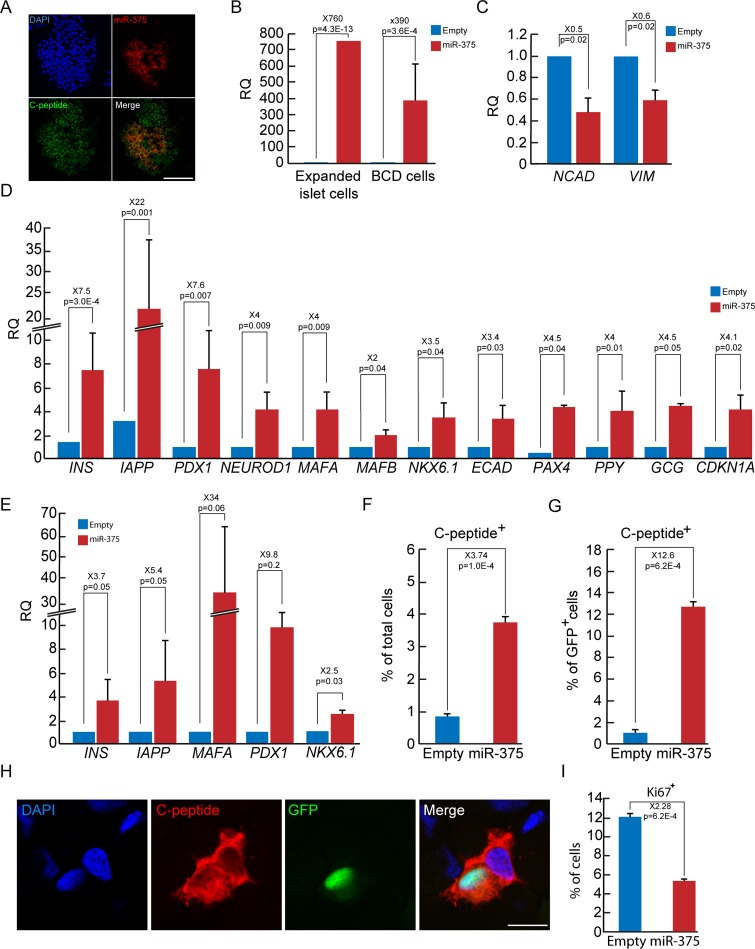
Effect of miR-375 overexpression on BCD cell redifferentiation. A. miR-375 *in-situ* hybridization in human islets. DNA was stained with DAPI. Bar = 75 μm. B. Overexpression of miR-375. Expanded islet cells, or sorted GFP^+^ BCD cells, at passages 5–12 were infected with miR-375 or empty viral vectors and analyzed 5 days later by qPCR. Data are mean±SE (for expanded islet cells, n = 8 donors; for BCD cells, n = 3 donors), relative to empty viral vector, and normalized to miR-24 and U6-snRNA. C, D. Changes in expression of mesenchymal genes (C) and islet cell genes (D) in expanded islet cells infected at passages 4–12 with miR-375 or empty viral vectors, and analyzed by qRT-PCR. Data are mean±SE (n = 4–8 donors). E. Changes in expression of β-cell genes in sorted GFP^+^ BCD cells infected at passages 4–7 with miR-375 or empty viral vectors, and analyzed 5 days later by qPCR. Data are mean±SE (n = 3 donors), relative to empty viral vector. F-H. Immunofluorescence analysis of C-peptide in expanded islet cells infected at passages 5–6 with miR-375 or empty viral vectors. F,G. Quantitation of C-peptide^+^ cells among total expanded islet cells (F), or GFP^+^ BCD cells (G). Values are mean±SD (n = 3 donors), based on counting >500 cells in each condition. H. Bar = 30 μm. I. Changes in proliferation of expanded islet cells infected at passages 3–5 with miR-375 or empty viral vectors, and analyzed 5 days later by immunofluorescence for Ki67. Values are mean±SD (n = 4 donors), based on counting >500 cells in each condition.

We used expression arrays to compare miRNA levels in expanded human islet cells following proliferation and dedifferentiation in culture, with those in isolated mature human islets. Twenty four miRNAs were downregulated (>2-fold) during the first two weeks of culture (equivalent to 2 passages), and 8 miRNAs were upregulated (Fig 1A). The miRNAs downregulated the most included the miR-141/miR-200 and miR-30 families, as well as miR-192, miR-204, and miR-215, which play key roles in maintaining epithelial cell phenotype [17,18], and their downregulation is in accordance with the EMT-like change occurring in cultured BCD cells. miR-7 participates in regulation of islet cell differentiation and function [19–21] and is abundant in mature islets [20,22], however its overexpression in developing pancreas inhibits α- and β-cell differentiation [19]. Downregulation of specific miRNAs was confirmed in sorted GFP^+^ BCD cells labeled by an insulin promoter-driven lineage tracing system [2] (Fig 1B). The expression array analysis demonstrated a >12-fold downregulation in miR-375 expression in expanded human islet cells, relative to islets. A much more pronounced (>380-fold) downregulation was revealed by qPCR in sorted GFP^+^ BCD cells, indicating a β-cell-specific effect. Furthermore, miR-375 was readily detected by in-situ hybridization in β cells of isolated adult human islets, and co-localized with C-peptide immunostaining (Fig 2A). In view of the important developmental and functional roles of miR-375 in β cells, we evaluated the effect of miR-375 overexpression on BCD cell redifferentiation.

### Effect of miR-375 overexpression on BCD cell redifferentiation

A pre-miR-375 retrovirus vector was used to overexpress pre-miR-375 and a bleomycin resistance gene in expanded BCD cells. miR-375 levels in bleomycin-resistant transduced cells were upregulated by several hundred-fold (Fig 2B) and were comparable to the expression levels of miR-375 in islet cells prior to dedifferentiation (Fig 1B). Restoration of miR-375 resulted in a 2-fold decrease in transcripts encoding the mesenchymal markers N-cadherin and vimentin (Fig 2C), and a 3-fold increase in E-cadherin mRNA levels (Fig 2D), as well as a change in cell morphology (S1 Fig), suggesting the induction of mesenchymal-epithelial transition (MET). mRNAs of several key β-cell transcription factors, including PDX1, MAFA, NKX6.1, NEUROD1, and PAX4, were upregulated 3.4–7.6-fold, and *INS* and *IAPP* transcripts were induced 7.5- and 22-fold, respectively. Consistent upregulation was observed in sorted GFP^+^ BCD cells (Fig 2E). In addition, miR-375 overexpression in expanded islet cells upregulated *MAFB* and *GCG* transcripts (Fig 2D). However, since the miR-375-induced *GCG*, *PPY*, and *SST* transcript elevation in sorted GFP^+^ BCD cells was insignificant (S2 Fig), we conclude that a distinct population of insulin-negative/glucagon-positive cells likely originates from non-BCD cells, in accordance with our previous results [5].

miR-375 further induced insulin protein formation, as judged by C-peptide immunofluorescence analysis (Fig 2F–2H). The vast majority (>98%) of C-peptide^+^ cells co-stained for PDX1 (S3 Fig). Redifferentiation efficacy approximated 12.5% of GFP^+^ cells (Fig 2G). This level of redifferentiation represents about half of that achieved with RC treatment [5]. In addition, redifferentiation was accompanied by a 4-fold increase in *CDKN1A* transcripts encoding the cell cycle inhibitor p21 (Fig 2D), and a >2-fold decrease in cell proliferation (from 12% to 5%), as judged by staining for Ki67 (Fig 2I). Minor changes in apoptosis rates were noted between uninfected cells (2.1±0.1%) and cells infected with miR-375 (2.5±0.2%) or empty viruses (S4 Fig). Taken together, these findings demonstrate that miR-375 induces profound changes in BCD cells and directs them towards redifferentiation.

### miR-375 overexpression in expanded islet cells downregulates the PDPK1-AKT pathway

**Fig 3:**
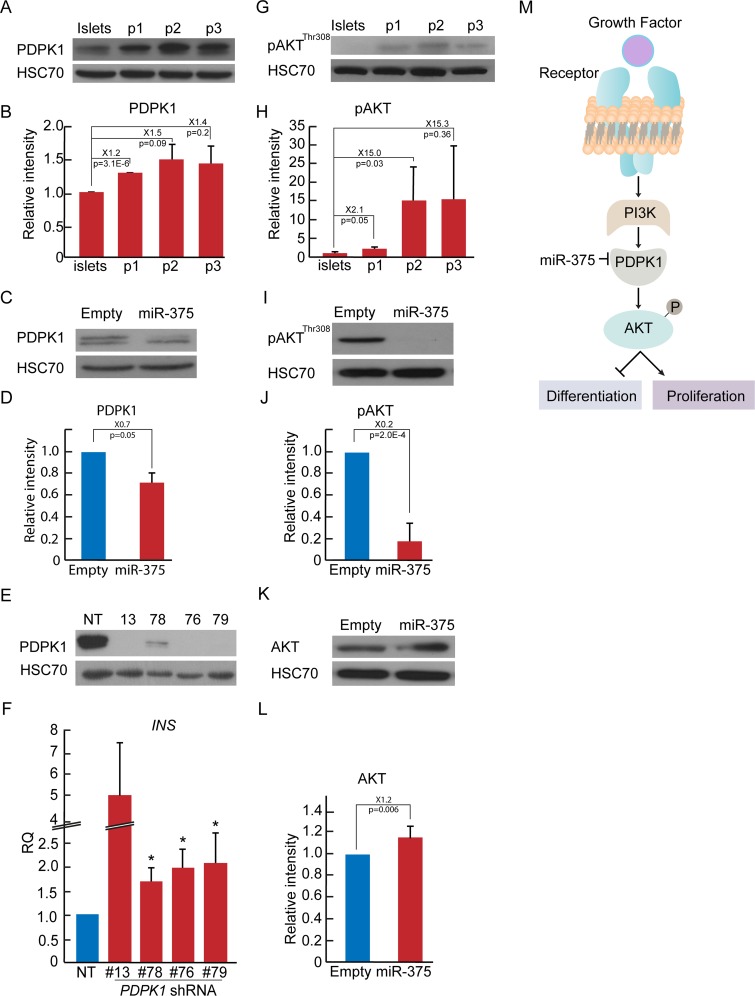
miR-375 overexpression in expanded islet cells downregulates the PDPK1-AKT pathway. A,B. Immunoblotting of PDPK1 in expanded islet cells at the indicated passages. HSC70 served as loading control. Values are mean±SE (n = 3 donors). C, D. Immunoblotting of PDPK1 in expanded islet cells infected at passages 4–6 with miR-375 or empty viral vectors. Values are mean±SE (n = 3 donors). E. Immunoblotting of PDPK1 in expanded islet cells at passages 4–6 infected with 4 different *PDPK1* shRNAs. NT, nontarget. F. Changes in insulin transcript levels in expanded islet cells infected at passages 4–6 with *PDPK1* or NT shRNAs, and analyzed 5 days later by qRT-PCR. Values are mean±SE (n = 3 donors), relative to NT. *p<0.05. G,H. Immunoblotting of phosphorylated AKT (Thr308) in expanded islet cells at the indicated passages. Values are mean±SE (n = 3 donors). I, J. Immunoblotting of phosphorylated AKT in expanded islet cells infected at passages 4–6 with miR-375 or empty viral vectors. Values are mean±SE (n = 3 donors). K,L. Immunoblotting of AKT in expanded islet cells infected at passages 4–6 with miR-375 or empty viral vectors. Values are mean±SE (n = 3 donors). *p = 0.006. M. Scheme of miR-375 effect on AKT targets through PDPK1.

To unravel the mechanism underlying these effects, we analyzed changes in expression of established and predicted miR-375 targets. While overexpression of miR-375 did not cause a significant change in the expression levels of *MTPN*, *HNF1B*, *PAX6*, and *NOTCH2*, a small but significant 18% decrease was detected in transcripts encoding 3-phosphoinositide dependent protein kinase-1 (PDPK1) (S5 Fig). PDPK1 is a serine-threonine kinase which mediates signaling downstream of PI3-kinase and is directly targeted by miR-375 [23]. As seen in Fig 3A and 3B, PDPK1 protein levels increased by 50% during the first 3 weeks of human islet cell expansion in culture, whereas miR-375 overexpression resulted in a significant 30% reduction in PDPK1 levels (Fig 3C and 3D). Knockdown of *PDPK1* by shRNAs was sufficient for induction of a significant increase in insulin transcripts in expanded islet cells (Fig 3E and 3F).

One of the main substrates of PDPK1 is AKT, which is activated by PDPK1-mediated Thr308 phosphorylation [24]. Dedifferentiation of BCD cells in the first three weeks of human islet cell expansion in culture was associated with a 15-fold elevation in phospho-AKT levels (Fig 3G and 3H). Accordingly, miR-375 overexpression resulted in a 5-fold reduction in phospho-AKT levels (Fig 3I and 3J), while total AKT protein levels slightly increased (Fig 3K and 3L). These findings position PDPK1 as an important functional target of miR-375 in a pathway that regulates BCD redifferentiation (Fig 3M).

### miR-375 overexpression downregulates GSK3

**Fig 4:**
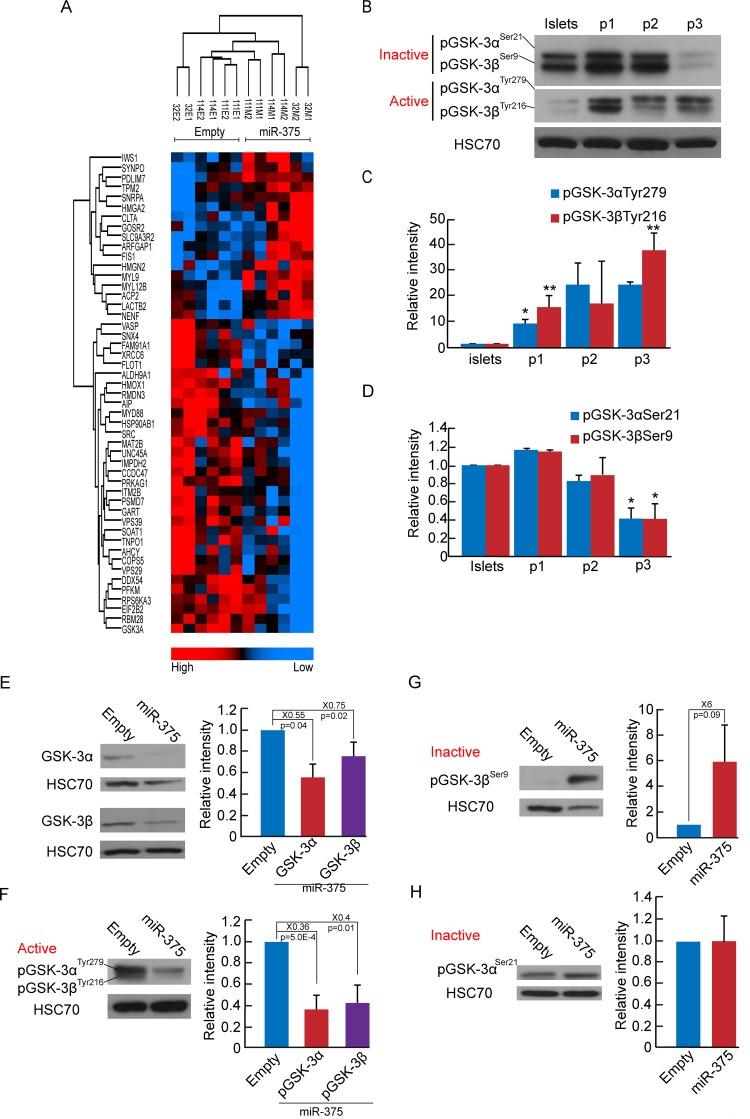
miR-375 overexpression downregulates GSK3. A. Proteomic profiling of sorted GFP^+^ BCD cells infected at passages 4–6 with miR-375 or empty viral vectors. Proteins changed >2-fold are shown. p≤0.05 (n = 3 donors, each analyzed in duplicates). B-D. Immunoblotting of phospohorylated GSK-3α and GSK-3β inactive or active forms in expanded islet cells at the indicated passages. Values are mean±SE (n = 3 donors). *p≤0.05; **p≤0.01. E. Immunoblotting of total GSK-3α and GSK-3β proteins in expanded islet cells infected at passages 3–5 with miR-375 or empty viral vectors. Values are mean±SE (for GSK-3α, n = 3 donors; for GSK-3β, n = 5 donors). F. Immunoblotting of active forms of GSK-3α and GSK-3β. Values are mean±SE (n = 6 donors). G. Immunoblotting of the inactive form of GSK-3β. Values are mean±SE (n = 4 donors). H. Immunoblotting of inactive form of GSK-3α in expanded islet cells infected at passages 3–4 with miR-375 or empty viral vectors. Values are mean±SE (n = 6 donors).

**Fig 5:**
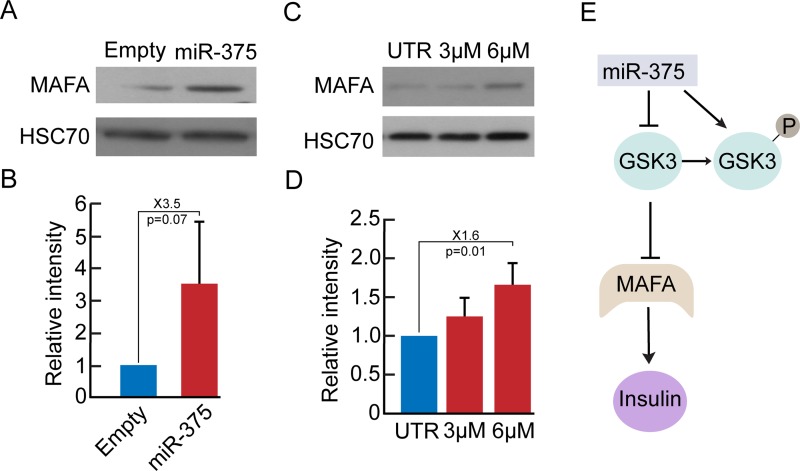
Downregulation of GSK3 is associated with MAFA upregulation. A,B. Immunoblotting of MAFA in expanded islet cells infected at passages 4–6 with miR-375 or empty viral vectors. Values are mean±SE (n = 3 donors). C,D. Immunoblotting of MAFA in expanded islet cells treated at passages 3–7 for 48h with the GSK-3 inhibitor SB-216763 at the indicated concentrations. Values are mean±SE (n = 3 donors). E. Suggested model for activation of insulin expression by miR-375-mediated inhibition of GSK3.

**Fig 6:**
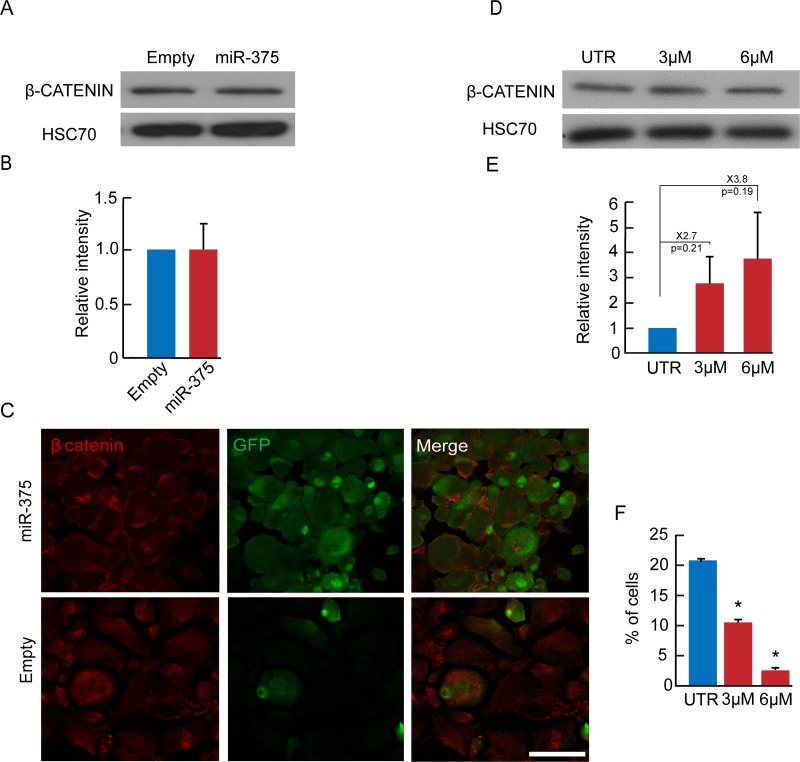
Changes in β-catenin and proliferation in expanded islet cells following miR-375 overexpression or GSK3 inhibitor treatment. A,B. Immunoblotting of cells infected at passages 4–6 with miR-375 or empty viral vectors. Values are mean±SE (n = 5 donors). C. Immunofluorescence of β-catenin in GFP-labeled expanded islet cells infected at passages 6 with miR-375 or empty viral vectors. Bar = 20 μm. D,E. Immunoblotting of cells treated at passages 3–7 for 48h with the GSK-3 inhibitor SB-216763 at the indicated concentrations. Values are mean±SE (n = 3 donors). F. Quantitation of Ki67 immunofluorescence in cells treated at passages 3–6 for 48h with the GSK-3 inhibitor SB-216763 at the indicated concentrations. Values are mean±SD (n = 4 donors). *p≤0.05.

Since target mRNA analysis may obscure miRNA effects manifested at the protein level, as a result of translation inhibition rather than transcript degradation, we performed mass spectrometric analyses for unbiased profiling of changes in gene expression induced in BCD cells by miR-375 overexpression. The analyses revealed changes in 49 proteins, 17 of them were upregulated, and 32 were downregulated (p<0.05) (Fig 4A). The protein downregulated to the largest extent was glycogen synthase kinase (GSK)-3α (1.6-fold difference between means of miR-375 overexpression and control). We therefore investigated changes in expression of both GSK-3α and GSK-3β during islet cell dedifferentiation and miR-375 overexpression. The levels of the active forms of both GSK-3α and GSK-3β were elevated >20-fold during the first 3 weeks of human islet cell expansion in culture, while the levels of the inactive forms decreased by 60% (Fig 4B–4D). miR-375 overexpression induced a significant decrease in total GSK-3α and GSK-3β protein levels (Fig 4E), as well as a decrease in the active forms of both proteins (Fig 4F). It also induced an increase in the inactive form of GSK-3β (Fig 4G), however levels of the inactive form of GSK-3α were not altered (Fig 4H). GSK-3α/β phosphorylates multiple substrates, including PDX1 [25,26] and MAFA [27], both of which are targeted for degradation following phosphorylation. MAFA protein levels were elevated in expanded islet cells overexpressing miR-375 (Fig 5A and 5B). To directly demonstrate that a reduction in GSK3 activity is involved in redifferentiation, we employed the GSK3 inhibitor SB-216763, which inhibits both GSK-3α and GSK-3β activity [28]. A 2-day treatment of expanded islet cells with SB-216763 induced a dose-dependent 60% increase in MAFA protein levels (Fig 5C and 5D). The decrease in GSK3 activity did not induce an increase in protein levels of β-catenin (Fig 6A and 6B), a key GSK3 target involved in regulation of cell proliferation [29]. Following miR-375 overexpression, β-catenin was predominantly localized near the plasma membrane, unlike control cells, in which it was detected throughout the cell (Fig 6C). As with miR-375 overexpression, SB-216763 did not increase total β-catenin protein levels (Fig 6D and 6E), and resulted in growth arrest (Fig 6F). Taken together, these findings suggest that GSK3 inhibition at least partially mediates the effect of miR-375 overexpression on BCD cell redifferentiation.

### Synergistic effects of miR-375 overexpression and RC on BCD cell redifferentiation

**Fig 7:**
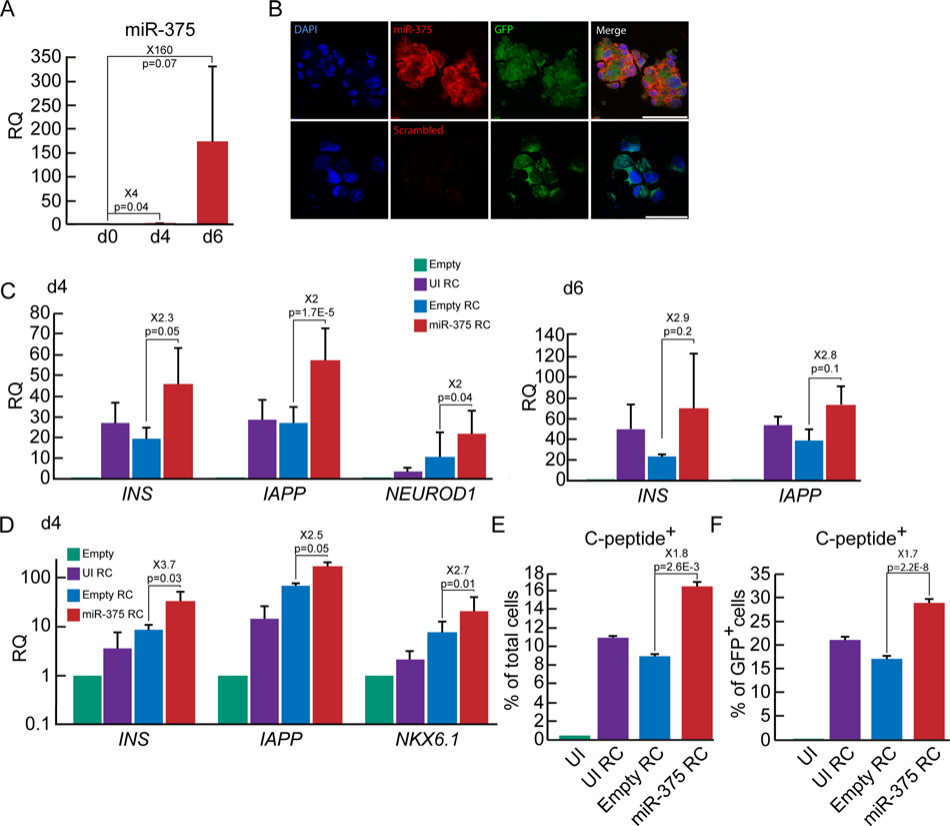
Synergistic effects of miR-375 overexpression and RC on BCD cell redifferentiation. A. qPCR analysis of changes in miR-375 levels in expanded islet cells treated at passages 5–7 with RC for the indicated times. Values are mean±SE (n = 3 donors), relative to d0, and normalized to miR-24 and U6-snRNA. B. *In-situ* hybridization with miR-375 or scrambled probe following a 4-day treatment with RC of expanded islet cells at passage 5 labeled with the β-cell lineage tracing vectors. DNA was stained with DAPI. Bar = 75 μm for miR-375, 50 μm for scrambled probe. C,D. qPCR analysis of changes in expression of β-cell genes in expanded islet cells (C) infected at passages 4–12, and sorted GFP^+^ BCD cells (D) infected at passages 4–7, with miR-375 or empty viral vectors and treated with RC for 4–6 days. Data are mean±SE (n = 3–9 donors in C, n = 3 donors in D), relative to cells infected with empty vector. UI, uninfected. E,F. Quantitation of immunofluorescence analysis of C-peptide in expanded islet cells infected at passages 5–6 with miR-375 or empty viral vectors and treated with RC for 4 days. Values are mean±SD (n = 3 donors), based on counting >500 cells in each condition.

We have previously shown that BCD cells can be redifferentiated by treatment with a combination of soluble factors in serum-free medium, termed Redifferentiation Cocktail (RC) [5]. RC treatment resulted in a detectable increase in miR-375 levels in expanded islet cells (Fig 7A) and GFP^+^ BCD cells (Fig 7B) during the first 4 days of treatment, and a further increase by 6 days (Fig 7A). Expanded islet cells subjected to a combined treatment of RC and miR-375 overexpression showed a 2-fold increase in β-cell transcripts, compared to RC treatment alone (Fig 7C). Similar results were obtained in sorted GFP^+^ BCD cells (Fig 7D). The combined treatment also resulted in a 70% increase in the number of C-peptide^+^ BCD cells, compared with cells treated with RC alone (Fig 7E and 7F). Overall, these findings suggest that increased miR-375 levels interact with the pathways activated by RC and result in enhanced BCD cell redifferentiation.

## Discussion

Our findings demonstrate that restoration of normal levels of a single miRNA, miR-375, in BCD cells is sufficient for induction of β-cell gene expression, reduced cell proliferation, and a switch from NCAD to ECAD expression, which is characteristic of mesenchymal-epithelial transition. These effects are reproducible in cells derived from multiple human donors. Our results support an important function of miR-375 in regulation of the differentiated human β-cell phenotype, and emphasize the roles of PDPK1 and GSK3 in mediating its effects.

*PDPK1* has been shown to be a direct target of miR-375 in rodent islet cells [23]. Our findings suggest that it is modulated by miR-375 in human islet cells as well. Analysis of the PDPK1-AKT pathway revealed a reduction of 30% in PDPK1 protein levels following miR-375 overexpression, resulting in 80%-decrease in phospho-AKT levels. Reduced activity of the PDPK1-AKT pathway may cause a decrease in BCD cell proliferation and an increase in cell differentiation. Indeed, mice deficient in PDPK1 in β cells manifest reduced β-cell numbers and hyperglycemia [30], while AKT overexpression under the *Pdx1* promoter results in β-cell dedifferentiation [31]. One possible mechanism by which a decrease in phospho-AKT activity may lead to growth arrest is by induction of p21 (*CDKN1A*; Fig 2D) [32].

Our findings implicate for the first time GSK3 in miR-375 activity in human islet cells. miR-375 overexpression downregulated GSK3α/β levels and activity, and upregulated the inactive form of GSK3β. Since *GSK3α* and *GSK3β* transcripts do not contain miR-375 binding sites, these likely represent indirect effects. Given that AKT is a negative regulator of GSK3β [33], the reduction in phospo-AKT would be expected to result in an increase in active GSK3β, and a decrease in inactive GSK3β. However, it is conceivable that additional protein kinases and phosphatases are involved in balancing the different phosphorylated states of GSK3 [34] following miR-375 overexpression. Our results suggest that a decrease in GSK-3β activity is associated with reduced islet cell proliferation. This is supported by the findings that miR-375 overexpression or the GSK3 inhibitor SB-216763 did not significantly increase β-catenin levels in expanded islet cells, and resulted in growth arrest. Apparently, the residual GSK3β activity is sufficient for regulating β-catenin levels. In contrast, β-cell-specific GSK-3β knockout in mice resulted in increased β-cell mass [35]. Nonetheless, GSK-3β has been associated with increased cell proliferation in other systems [36,37].

miR-375 overexpression in expanded islet cells resulted in MET, as judged by downregulation of N-cadherin and upregulation of E-cadherin. Recent work has identified SHOX2, an inducer of EMT in breast cancer cells, as a novel miR-375 target [38], suggesting a possible mechanism for restoration of the epithelial phenotype in BCD cells by miR-375.

Expression of miR-375 by itself induced detectable C-peptide expression in only 12% of BCD cells, making it difficult to assess cell function, such as glucose-stimulated insulin secretion (GSIS). However, miR-375 overexpression synergized with RC treatment in promoting BCD cell redifferentiation, as manifested by a 3.7-fold increase in insulin transcript levels, and a 1.8-fold increase in the number of C-peptide-positive cells, compared with RC alone, which were highly reproducible in cells from multiple human donors. This synergy occurred despite the significant miR-375 upregulation induced by RC alone, suggesting a quantitative correlation between miR-375 levels and insulin expression in BCD cells. miR-375 has been implicated in limiting GSIS under stress in MIN6 cells by downregulating myotrophin (*Mtpn*) transcripts [8]. However, the levels of human *MTPN* mRNA did not change following miR-375 overexpression in expanded islet cells, and miR-375 upregulation by RC did not inhibit GSIS in BCD cells [5].

Our results suggest that miR-375 expression may contribute to future approaches for cell replacement therapy of diabetes based on in-vitro expansion and redifferentiation of donor islet cells. Clinical application will require non-viral delivery of miR-375, functional assessment of the redifferentiated cells in vitro and in vivo, and development of effective immunoprotective approaches.

## Supporting Information

S1 Fig Morphological changes in expanded islet cells at passage 6, 12 days following infection with miR-375 viral vector. UTR, untreated. Phase contrast images. Bar = 400 μm.
(TIF)

S2 Fig Changes in expression of hormone genes in BCD cells. RNA was extracted from sorted GFP^+^ BCD cells 5 days following infection at passages 4–7 with miR-375 or empty viral vectors, and analyzed by qPCR. Data are mean±SE (n = 3 donors), relative to empty viral vector.
(TIF)

S3 Fig Immunofluorescence analysis of C-peptide and PDX1 in GFP-labeled expanded islet cells infected at passage 5 with miR-375 or empty viral vector. Bar = 20 μm.
(TIF)

S4 Fig Changes in apoptosis rates in expanded islet cells infected at passages 3–5 with miR-375 or empty viral vectors, and analyzed 5 days later by TUNEL. Values are mean±SD (n = 3 donors), based on counting >500 cells in each condition.
(TIF)

S5 Fig Changes in expression of established and predicted miR-375 targets in expanded islet cells infected at passages 4–12 with miR-375 or empty viral vectors, and analyzed by qRT-PCR. Data are mean±SE (n = 3–6 donors).
(TIF)
