# Supplementary figures and images for: MiR-375 Promotes Redifferentiation of Adult Human β Cells Expanded In Vitro
Source: PLoS One. 2015 Apr 13;10(4):e0122108. doi: 10.1371/journal.pone.0122108 (PMC4395232; doi:10.1371/journal.pone.0122108)

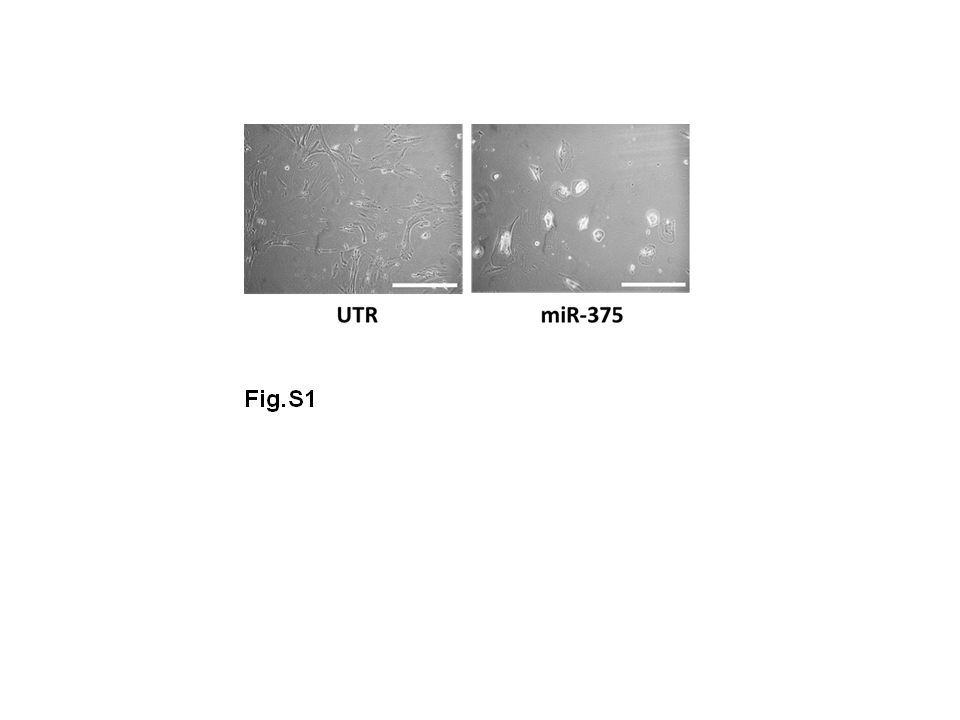

Supplement: S1 Fig — UTR, untreated. Phase contrast images. Bar = 400 μm. (TIF) [file pone.0122108.s001.TIF]

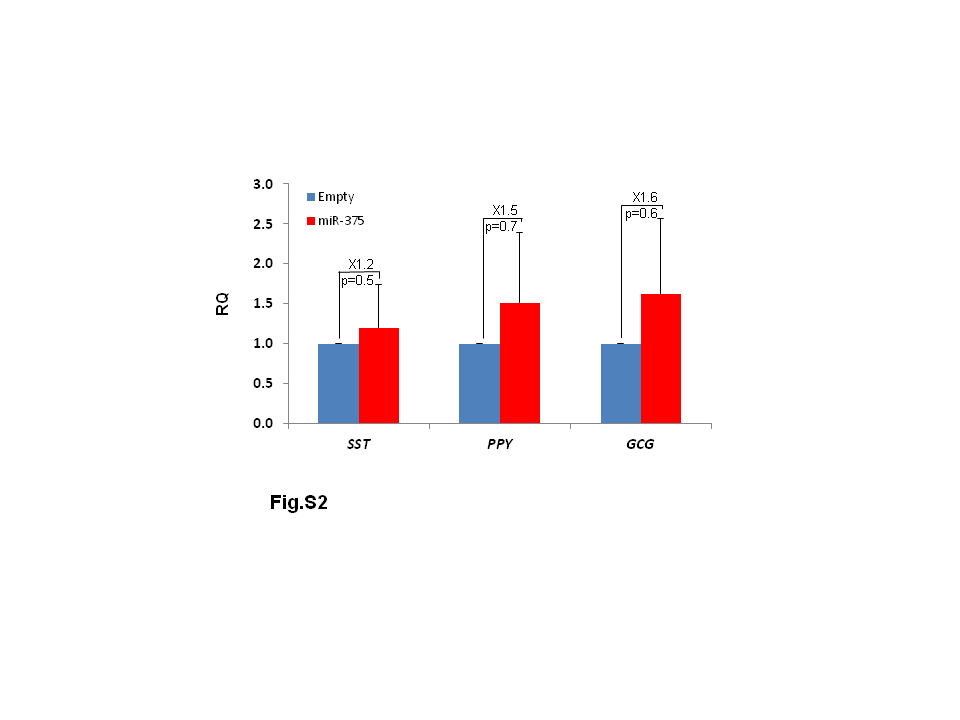

Supplement: S2 Fig — RNA was extracted from sorted GFP+ BCD cells 5 days following infection at passages 4–7 with miR-375 or empty viral vectors, and analyzed by qPCR. Data are mean±SE (n = 3 donors), relative to empty viral vector. (TIF) [file pone.0122108.s002.TIF]

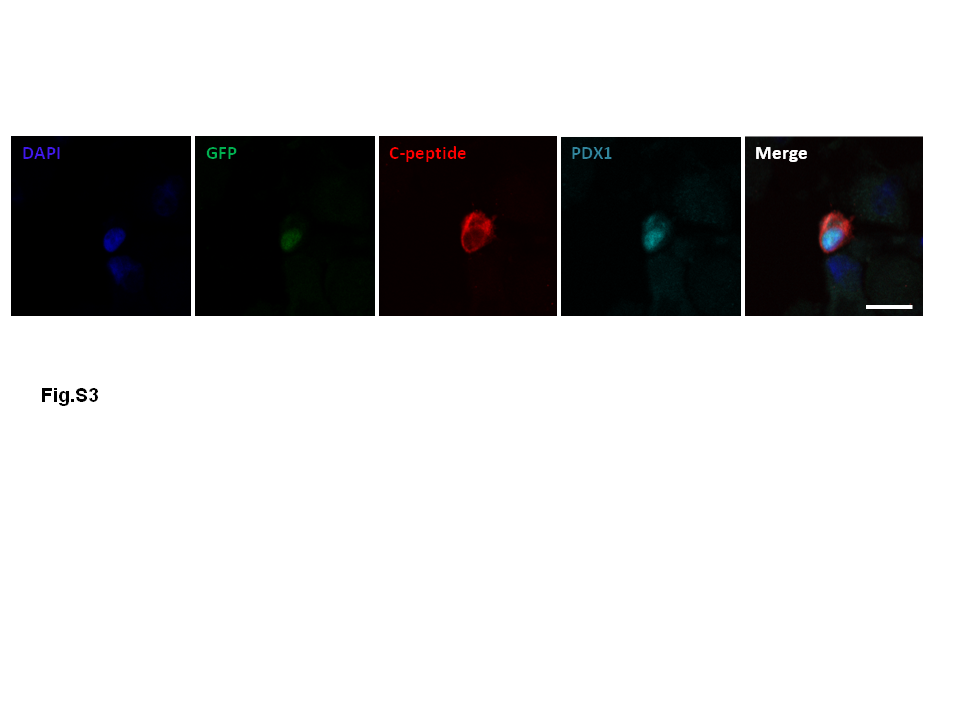

Supplement: S3 Fig — Bar = 20 μm. (TIF) [file pone.0122108.s003.TIF]

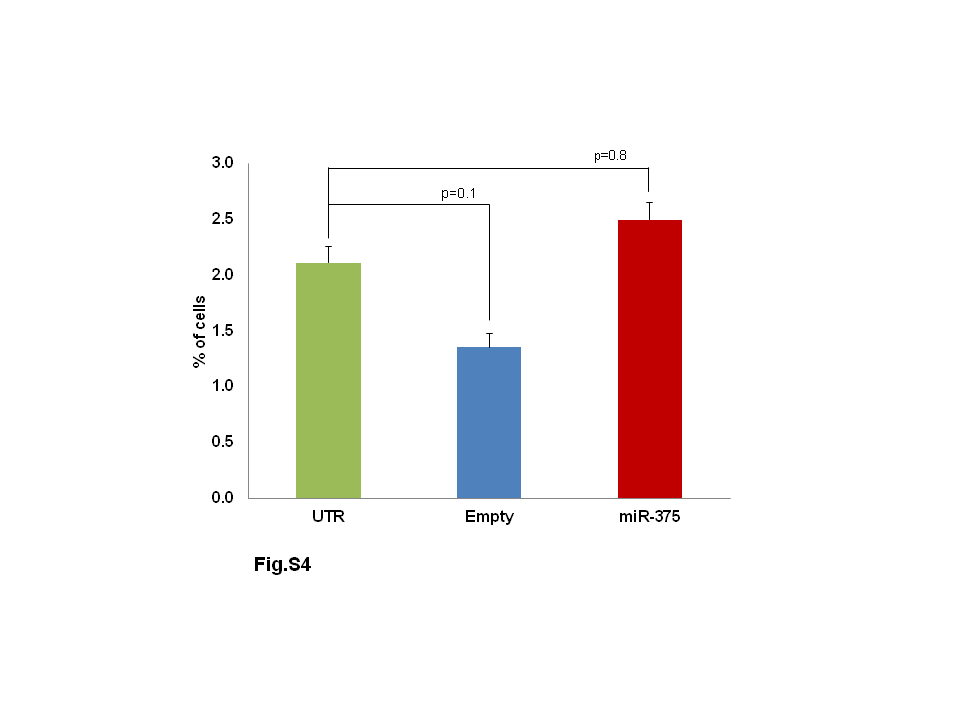

Supplement: S4 Fig — Values are mean±SD (n = 3 donors), based on counting >500 cells in each condition. (TIF) [file pone.0122108.s004.TIF]

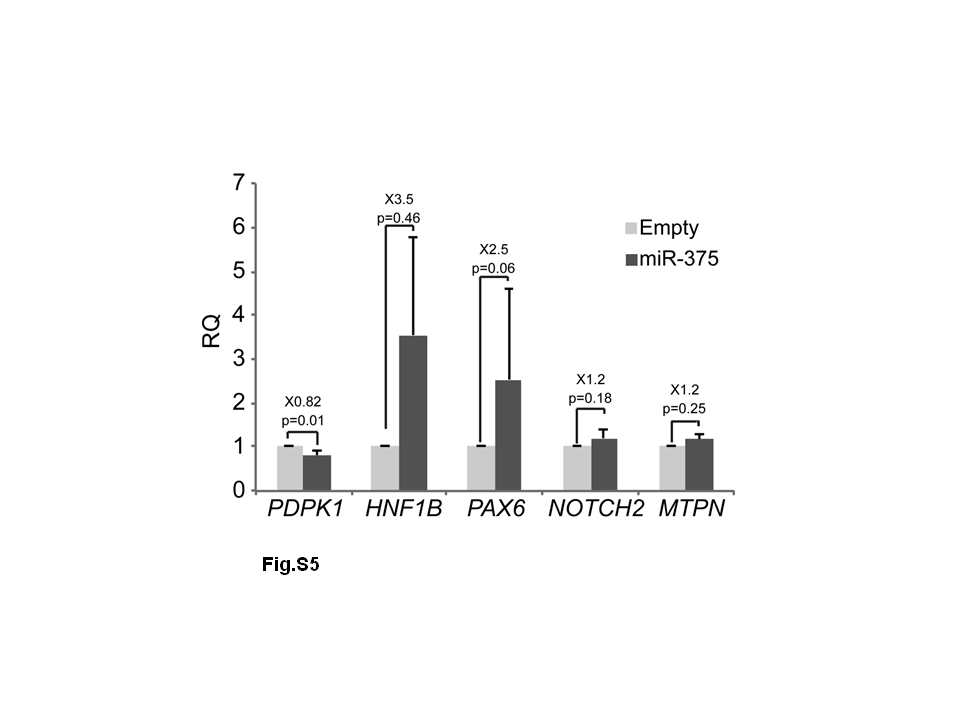

Supplement: S5 Fig — Data are mean±SE (n = 3–6 donors). (TIF) [file pone.0122108.s005.TIF]
